# Development to metamorphosis of the nemertean pilidium larva

**DOI:** 10.1186/1742-9994-7-30

**Published:** 2010-12-02

**Authors:** Svetlana A Maslakova

**Affiliations:** 1 Oregon Institute of Marine Biology, University of Oregon, Charleston, OR 97420, USA

## Abstract

**Background:**

The nemertean pilidium is one of the most notable planktotrophic larval types among marine invertebrates. The juvenile forms inside the larva from a series of isolated rudiments, called the imaginal discs. The development culminates in catastrophic metamorphosis, in which the larval body is consumed by the juvenile worm. Although the pilidium was first described in 1847, and is commonly found among marine plankton, there is not a single complete description of its development. The few published studies of pilidial development are based on observations of typically unidentified larvae opportunistically collected from plankton at various developmental stages.

**Results:**

The development of *Micrura alaskensis*, a common Northwest Pacific coast intertidal nemertean, is described from fertilization to metamorphosis. A staging scheme is proposed based on characteristic developmental milestones. Three pairs of imaginal discs develop as invaginations of larval epidermis. The cephalic discs invaginate from the larval epidermis above the ciliated band, while the cerebral organ discs and the trunk discs invaginate below the ciliated band. All paired imaginal disc invaginations are closely associated with different portions of the larval ciliated band. In addition, two unpaired rudiments contribute to the juvenile - the proboscis rudiment and the dorsal rudiment, which do not develop as invaginations. A pair of thick-walled esophageal pouches previously thought to represent nephridial rudiments give rise to the juvenile foregut. Branched rudiments of protonephridia, and their efferent ducts are also described. Larval and juvenile serotonergic nervous systems are briefly described. Development of the juvenile is completed by 5-8 weeks at 11-15 degrees C. During the rapid metamorphosis the juvenile emerges from and devours the larva.

**Conclusions:**

This study is the first description of pilidial development from fertilization to metamorphosis in a single species. It is illustrated with photomicrographs of live larvae, diagrams, confocal images, and videos. The findings are discussed in the context of previously published accounts of pilidial development, with which they disagree on several accounts. The results described here indicate a different number, origin and fate of various juvenile rudiments. The proposed staging scheme will be useful in subsequent studies of pilidial development.

## Background

The ciliated planktotrophic pilidium larva of nemertean worms (phylum Nemertea) is one of the most characteristic and unmistakable representatives of marine plankton. It is shaped like a helmet and equipped with an apical tuft of long cilia, and, typically, four lobes, spanned by the larval ciliated band [1]. The thin-walled and funnel-like pilidial esophagus (technically a vestibule) is separated from the thick-walled globose stomach by a sphincter (which could be considered as the pilidial mouth). The larval gut ends blindly. The larva swims with the apical organ pointing forward, and the larval antero-posterior axis corresponds to the animal-vegetal axis of the zygote. The pilidium larva, first described by Müller in 1847 [2], looks nothing like the juvenile nemertean worm, which Metschnikoff discovered in 1869 [3] develops inside the larva from a series of isolated rudiments called the imaginal discs. In a typical pilidium the antero-posterior axis of the juvenile is at a 90° angle with respect to the antero-posterior axis of the larva. The unusual development of the pilidium culminates in a catastrophic metamorphosis during which the juvenile worm emerges from and devours the larval body [4,5]. Although the term "metamorphosis" has been applied, particularly in the older literature, to the entire process of development of the juvenile worm inside the pilidium larva, I am using it in a more restrictive sense, referring to the end-point of pilidial development - i.e. the rapid transition between the pilidium and the juvenile.

The pilidium larva is found in a single clade called the Pilidiophora [6], which comprises roughly a third of all nemertean species [7], and represents a derived mode of development for the phylum [8]. Other nemerteans possess uniformly ciliated planula-like planktotrophic or lecithotrophic larvae, or direct development [1,9,10]. Pilidia belonging to different species vary in shape, size and color, but few have been matched to their respective adults [5,10-12]. Modifications of pilidial development include Desor's larva in *Lineus viridis *[13] and Schmidt's larva in *Lineus ruber *[14], Iwata's larva in *Micrura akkeshiensis *[15] and a few recently discovered others [[10,16], Megan Schwartz (University of Puget Sound) pers. comm.]. Development of Desor's and Schmidt's larva is encapsulated, while the others have planktonic lecithotrophic development. In all of these cases, the pilidial lobes are lacking, the blastocoel is reduced, the larva does not feed, but, similar to the canonical pilidium the juvenile develops via imaginal discs.

Although the development of the juvenile from imaginal discs is surely the most remarkable feature of the pilidium, this process remains poorly understood. The larval epidermis is very thin and transparent, making it easy to observe juvenile rudiments in live larvae. However, because the larvae are planktotrophic and the development of the juvenile takes weeks to months, the few studies of pilidial development are limited to descriptions of early stages, i.e. before onset of rudiment formation [12,17-20], or observations based on unidentified pilidium larvae opportunistically collected from plankton [21-23]. The only published report of pilidium larvae raised from fertilization to the point of formation of a nearly complete set of imaginal discs is that by Schmidt [24] for *Cerebratulus marginatus *(see [25] for German translation). Most of what we know about the development of the juvenile inside the pilidium comes from the 1912 study by Salensky [23].

On the other hand, the development of the juvenile inside the encapsulated Desor's "larva" has been studied in detail by many authors [13,26-29]. These embryos are opaque, so the observations are based on studies of preserved and sectioned material. Because this mode of development is derived for Pilidiophorans, it is not clear how much of what is known about the juvenile development inside Desor's larva is peculiar to it, and how much pertains to the pilidial development. For example, Desor's larva is described to have a total of eight imaginal discs, including a separate proboscis rudiment, whereas the typical pilidium is considered to have a total of seven discs, with proboscis developing as a derivative of the cephalic discs [[23] and references therein, and modern textbooks, e.g. [1,9,30]], although see Bürger [22] and Schmidt [24,25].

This paper describes the development of a typical pilidium larva in *M. alaskensis *from fertilization to metamorphosis, focussing particularly on the relative timing of developmental events, and the number and origin of the various juvenile rudiments. A staging scheme is proposed based on characteristic developmental milestones. Larval and juvenile serotonergic nervous systems are briefly described. The results are discussed in the light of previously published accounts of pilidial development.

## Results

### Developmental timeline

Using the method described here, I successfully reared to metamorphosis dozens of cohorts of pilidium larvae of *Micrura alaskensis*.

**Figure 1:**
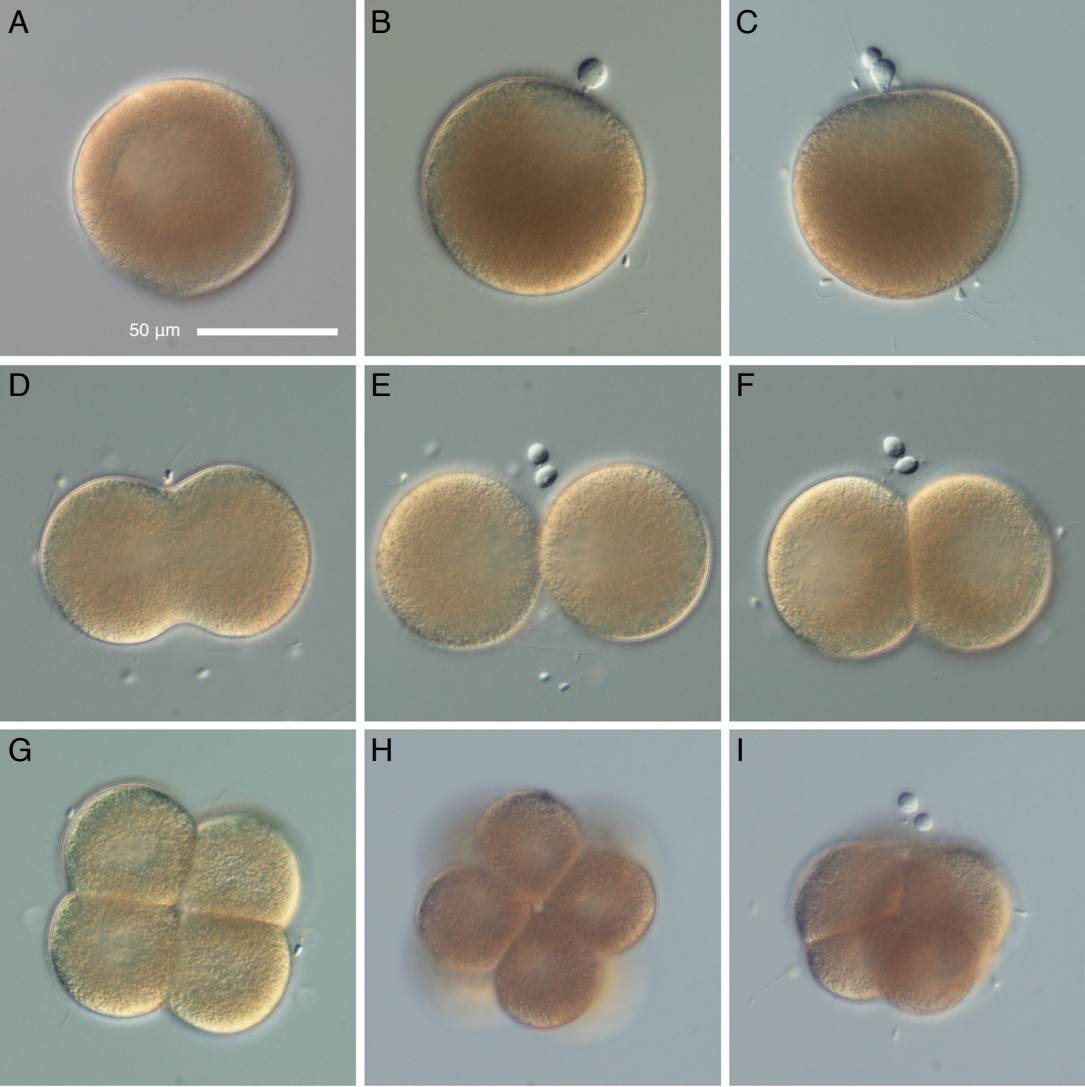
**Early cleavage in *Micrura alaskensis***. DIC images. A. Unfertilized egg undergoing germinal vesicle breakdown. B. Fertilized egg with the first polar body. C. Formation of the second polar body. D-F. First cleavage. D. Broad initial cleavage furrow. E. There is no egg chorion, and the blastomeres separate almost completely. F. Embryo re-compacts after first cleavage. G. Polar view of the four-cell stage. H. Vegetal view of an eight-cell stage illustrating dextral spiral cleavage. I. Side view of an eight-cell stage. Apical pole, marked by polar bodies, is up. Animal micromeres are larger than the vegetal macromeres.

Dissected oocytes are frisbee-shaped and possess conspicuous germinal vesicles. They round up and undergo germinal vesicle breakdown within 30-40 min of contact with sea water. The round eggs are relatively opaque, 76 μm in diameter on average (n = 13), without a distinct egg envelope (Figure 1A). The transparent layer of jelly surrounding the eggs is easily removed by pipetting or repeatedly passing the eggs through a nitex mesh with mesh size slightly larger than the egg diameter. As in other nemerteans, spiral cleavage is equal and holoblastic (Figure 1A-I). At 11°C the first polar body is formed within the first 20-30 minutes (Figure 1B), and the second polar body forms at about 1 hour after fertilization (Figure 1C). The first cleavage occurs at about 2 hours 15 min after fertilization (Figure 1D-F), and the subsequent divisions occur about once an hour. The initial cleavage furrow is broad (Figure 1D) and the blastomeres separate almost completely (Figure 1E), then re-compact (Figure 1F). The third division is dextral (Figure 1H) and, as in other nemerteans, the first-quartet micromeres are slightly larger than the macromeres in the 8-celled embryos (Figure 1I).

**Figure 2:**
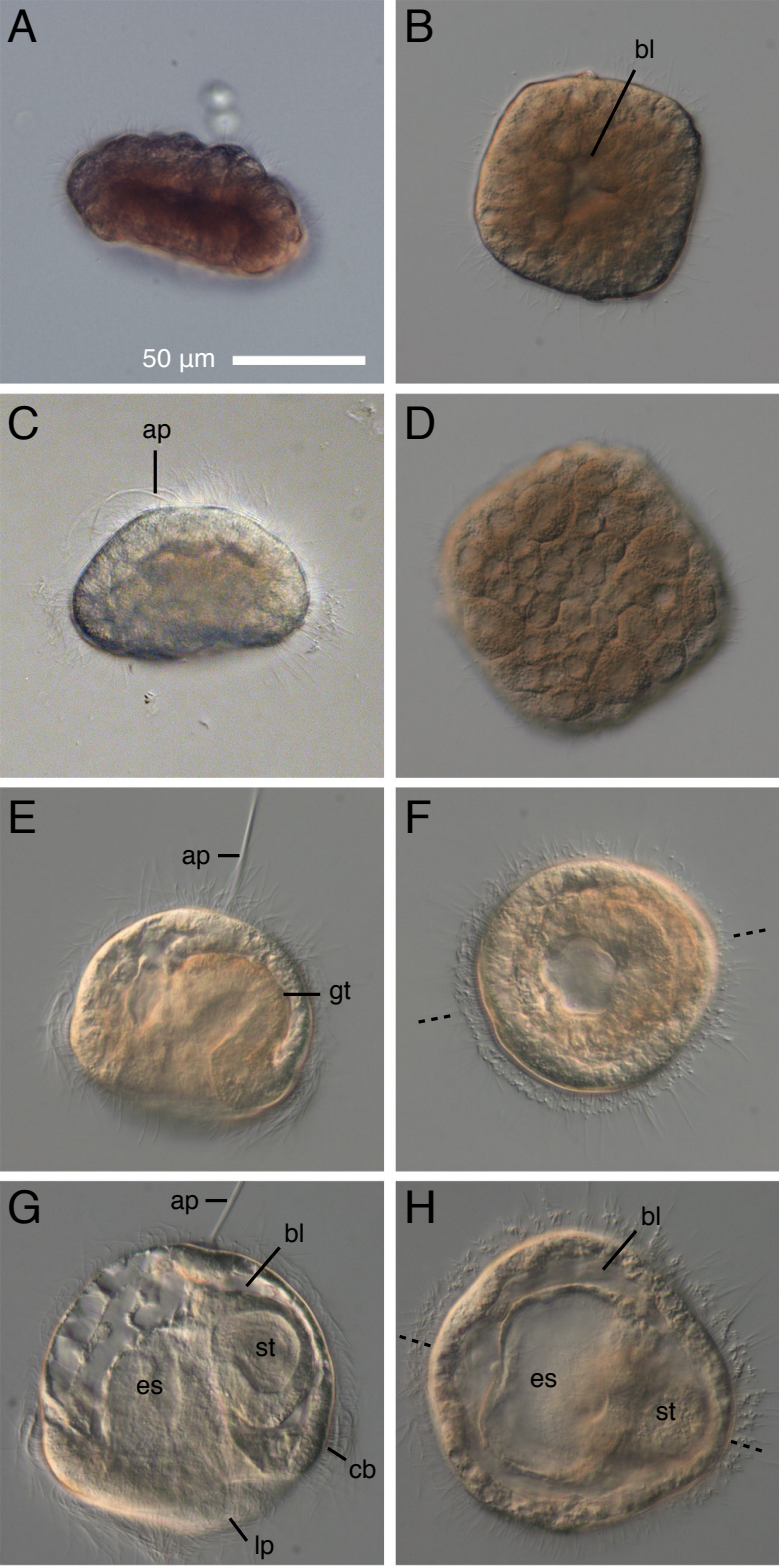
**Development of *Micrura alaskensis *from blastosquare to young pilidium**. DIC images. Left panels - side view, right panels - polar view. A-B, D. Ciliated blastosquare (18-20 hours after fertilization). Note the small blastocoel (bl) on B and spiral cleavage pattern apparent in a slightly compressed blastosquare (D). C. Gastrula with an apical tuft (ap) (27-hours-old). E-F. Two-day-old pilidium (49-53-hours-old) showing bilaterally symmetrical gut (gt) and a long apical tuft. Plane of bilateral symmetry is indicated by dashed line on F and H. G-H. Three-day-old pilidium has a blind gut differentiated into esophagus (es) and stomach (st), a prominent apical tuft, ciliated band (cb), and nascent lateral lappets (lp). The young pilidium increases in size even before onset of feeding due to the expansion of the blastocoel.

**Figure 3:**
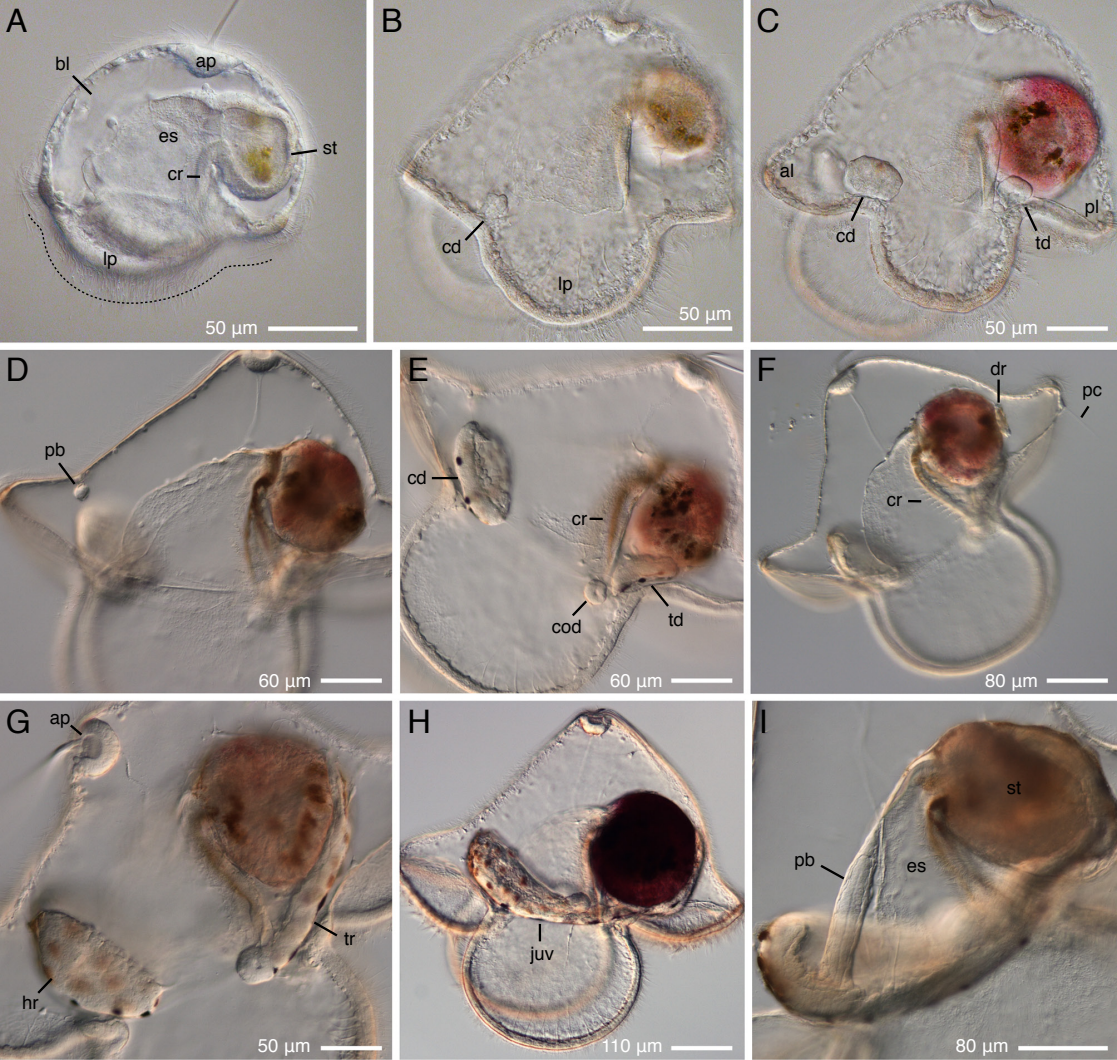
**Onset of feeding, and early juvenile development in *Micrura alaskensis***. DIC images. Lateral views. Apical plate up, juvenile anterior left. A. Four-day-old feeding pilidium features an apical plate (ap), extensive blastocoel (bl), esophagus (es) with a pair of ciliated ridges (cr), stomach (st), and circumoral ciliated band (dotted line) spanning the lappets (lp). B. The cephalic imaginal discs (cd) appear in vicinity of the ciliated band at the anterior margin of the lateral lappets after about one week of development. Stomach is colored with pigment derived from algal cells. C. The second pair of imaginal discs, called trunk discs (td), appears in vicinity of the ciliated band at the posterior margin of the lateral lappets as early as 9 days of development. At this stage the anterior larval lobe (al) and posterior lobe (pl) are well developed. D-E. The third pair of imaginal discs, the cerebral organ discs (cod), and the unpaired proboscis rudiment (pb) appear nearly simultaneously, as early as 15-17 days of development. The cerebral organ discs invaginate from the inner surface of the lateral lappets near the esophageal ciliated ridges (cr). F. The unpaired dorsal rudiment (dr) appears between the stomach and the pilidial epidermis in diametrically opposite position to the proboscis rudiment as early as 24 days of development. Note the pilidial posterior cirrus (pc). G. Head and trunk stage. The cephalic discs fuse with each other and the proboscis rudiment to form the head rudiment (hr). The cerebral organ discs together with the trunk discs and the dorsal rudiment form the trunk rudiment (tr). H. Torus stage is characterized by a doughnut of juvenile tissue (juv) around the larval gut formed by the fused head and trunk rudiments. I. Extended proboscis stage is characterized by the proboscis rudiment growing out of the forming juvenile head along the larval esophagus and reaching beyond the head margin.

The blastula of *Micrura alaskensis *is flattened along the animal-vegetal axis, and square-shaped, hence I refer to it as a "blastosquare" (Figure 2A-B, D). The blastosquare has a small blastocoel (Figure 2B). Blastosquares become ciliated and start swimming as early as 16 hours after fertilization at ambient sea temperature (11-14°C). By 24 hours of development swimming gastrulae are radially symmetrical, possess an apical tuft at the animal pole, and a prominent invagination at the vegetal pole (Figure 2C). By the end of the second day of development the young pilidium larva exhibits bilateral symmetry, as the developing gut is inclined toward the dorsal side (Figure 2E-F). The young helmet-like pilidium larva is equipped with a long apical tuft, two lateral lappets, and a band of longer cilia spanning the lappets. The gut of the pilidium larva is a blind sack differentiated into the funnel-like thin-walled esophagus (or vestibule) and the thick-walled stomach, with a muscle sphincter in between (Figure 2G-H). The posterior wall of the esophagus (with respect to the axis of the future juvenile worm) has a pair of ciliated ridges (Figure 3A). The pilidia are capable of feeding on unicellular algae as early as 66 hours after fertilization. The stomachs of pilidia feeding on *Rhodomonas lens *gradually accumulate brown-magenta pigment (Figure 3). As the pilidia grow the lateral lappets become more and more pronounced, and two additional lobes develop, which are usually referred to as the anterior and the posterior (with respect to the juvenile-to-be) (Figure 3C).

The rate of development depends on the temperature, feeding regime, and, possibly, other factors. Development of *M. alaskensis *in culture is somewhat asynchronous, even among full siblings raised in the same container. However, the juvenile rudiments always appear in the same sequence: first a pair of cephalic discs (Figure 3B), then a pair of trunk discs (Figure 3C), followed by the unpaired proboscis rudiment (Figure 3D), a pair of cerebral organ discs (Figure 3E), and the unpaired dorsal rudiment (Figure 3F). I observed two pairs of imaginal discs, the cephalic and the trunk discs, as early as 9 days after fertilization, although, more typically, at 14-15 days. The proboscis rudiment appears at about the same time as the cerebral organ discs, as early as 14 days of development, but more typically around 20 days.

**Figure 4:**
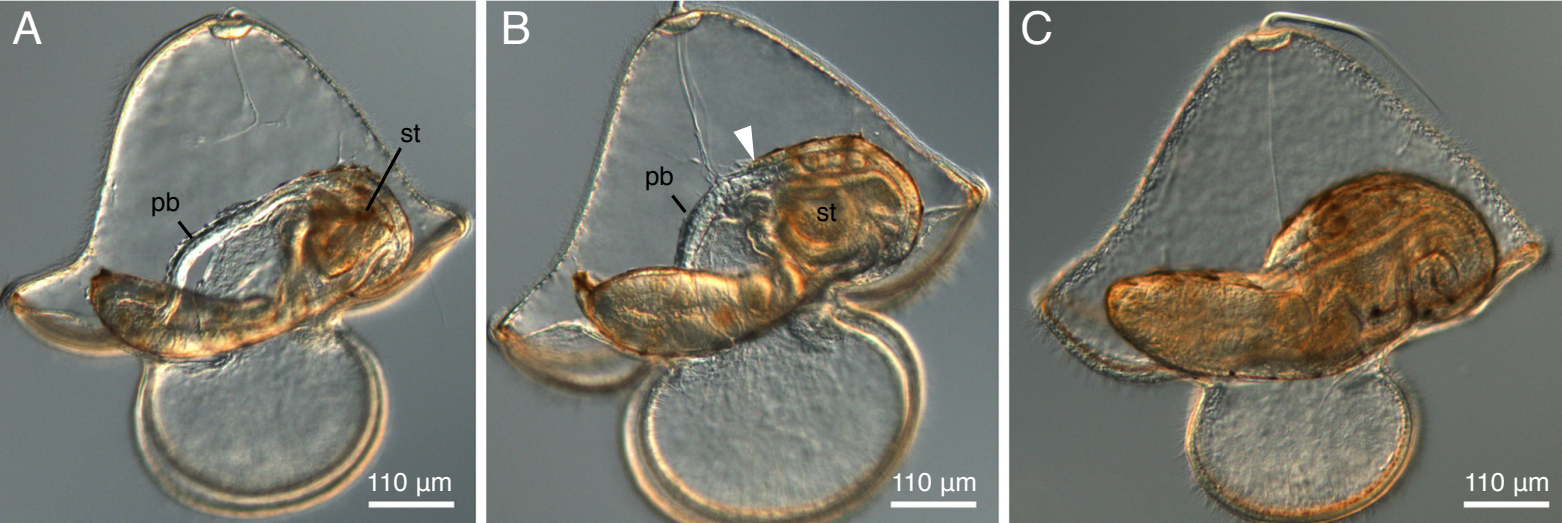
**Advanced stages of juvenile development inside pilidium of *Micrura alaskensis***. DIC images. Lateral views. Apical plate up, juvenile anterior left. A. Complete proboscis stage is characterized by proboscis (pb) reaching the stomach (st). B. Hood stage is characterized by the anterior margin of the juvenile dorsal epidermis overlapping the proboscis (arrowhead). C. A complete juvenile inside the pilidium larva. At this stage the larva is competent to undergo metamorphosis.

The proboscis rudiment fuses with the cephalic discs, forming the head rudiment, and the cerebral organ discs fuse with the trunk discs (Figure 3G). About the same time, a small dorsal rudiment appears just below the stomach and dorsal to the trunk discs (Figure 3F). It is initially clearly separate, and later fuses with the trunk discs contributing to the trunk rudiment. Subsequently, the head rudiment fuses with the trunk rudiment on both sides forming a toroid juvenile rudiment (Figure 3H). This "torus" stage was observed as early at 28 days after fertilization, although development to this point may take 5 weeks or longer. The proboscis rudiment elongates and reaches beyond the margin of the juvenile head, stretching out along the dorsal side of larval esophagus toward the stomach (Figures 3I, 4A). The dorso-posterior wall of the juvenile rudiment gradually grows over the larval stomach and overlaps the proboscis. At this stage the juvenile rudiment most resembles a slipper shoe with a hole in the middle of its "sole" (Figure 4B). The edge of the dorsal wall continues to grow anteriorly, covering up the proboscis, and eventually it fuses with the margin of the head rudiment, thus closing the dorsal gap and completing the juvenile (Figure 4C).

**Figure 5:**
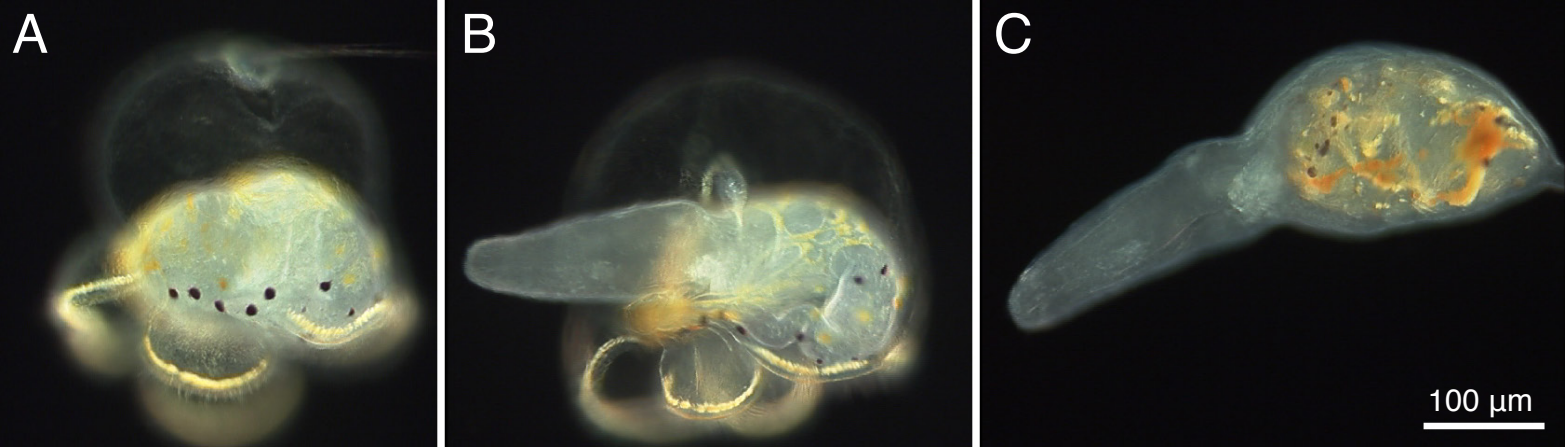
**Metamorphosis of the pilidium larva of *Micrura alaskensis***. Frames from a dark field microscopy video. A. Juvenile inside an intact pilidium larva. Golden pigment granules highlight the larval ciliated band. The juvenile of this species is enclosed within a thin amnion characteristically decorated with black and light brown pigment granules. B. The juvenile head ruptured through and emerged from the amnion and the pilidial epidermis. C. Newly metamorphosed juvenile devoured the entire larval body, as is evident from the pilidial pigment granules inside the gut showing through the body wall. See also additional file 1 -- Movie 1.

Metamorphosis of cultured pilidia of *M. alaskensis *occurred as early as 35 days after fertilization, but more typically at 6-7 weeks. Pilidium larvae with complete juveniles readily underwent metamorphosis in culturing containers, and even on microscope slides, trapped under coverglass while being filmed (Figure 5, Additional file 1 -- Movie 1). In the vast majority of cases, the emerging juvenile devoured the entire larval body (Figure 5C, Additional file 1 -- Movie 1).

Because development of pilidia in culture is typically asynchronous, it makes little sense to refer to the larval stages by absolute age. To facilitate future studies of pilidial development in this and other nemertean species, I propose a staging scheme based on the presence of certain developmental milestones (Table 1).

**Table 1:** Description of larval stages of the pilidium of Micrura alaskensis based on certain developmental milestones.

Stage	Developmental milestones	Earliest age (PF) observed in culture
blastosquare	flat square blastula, swims with cilia (Figure 2A-B, D)	16 hours

gastrula	radially symmetrical swimming stage with an apical tuft and a vegetal invagination (Figure 2C)	24 hours

young pilidium	bilateral symmetry apparent in the gut, longer cilia along the vegetal margin, nascent lateral lappets (Figure 2E-H)	40 hours

feeding pilidium	helmet-shaped pilidium with a ciliated band spanning the lappets, capable of capturing unicellular algae (Figure 3A)	66 hours

cephalic discs	a pair of cephalic discs is present (Figures 3B, 6)	7 days

trunk discs	a pair of trunk discs is present (Figure 3C, 7)	9 days

cerebral organ discs	a pair of cerebral organ discs appears near the trunk discs at the same time as the proboscis rudiment (Figures 3D-E, 8)	14 days

head and trunk	the proboscis rudiment fused with the cephalic discs to form the head rudiment; cerebral organ discs fuse with the respective trunk discs to form the trunk rudiment; an unpaired dorsal rudiment appears near the larval stomach, it is initially separate from the trunk discs and later fuses with them (Figure 3F-G, 9E)	24 days

torus	head and trunk rudiments fuse forming a toroid of tissue around the larval gut (Figure 3H, 9F)	28 days

extended proboscis	proboscis rudiment extends beyond the dorsal margin of the juvenile head, but has not yet reached the stomach (Figures 3I, 9G)	NA

complete proboscis	proboscis reaches the stomach and contacts the dorsal margin of the juvenile trunk epidermis (Figures 4A, 9H)	NA

hood	dorsal margin of the juvenile trunk extended over the posterior portion of proboscis, but has not yet reached and fused with the dorsal margin of juvenile head (Figure 4B)	NA

metamorphosis	fully formed juvenile emerges from the larva and devours it (Figure 5, see also additional file 1 - Movie 1)	35 days

### Development of the juvenile rudiments

**Figure 6:**
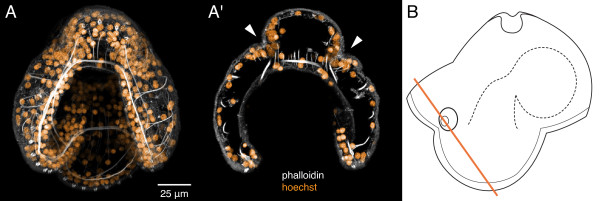
**Cephalic disc invaginations in a 7-day-old pilidium of *Micrura alaskensis***. A, A' Confocal images of the pilidium larva stained with phalloidin (white) and hoechst (orange). Anterior up. A. A z-projection of the entire larva, looking into the mouth. A'. A sub-stack (8 μm thick) of the same larva, which highlights the paired cephalic disc invaginations (arrowheads), which originate from the outer epidermis of the pilidium i.e. above the ciliated band. B. A diagram of the pilidium larva in lateral aspect (anterior to the left) illustrating the plane of sectioning (orange line). See also additional file 2 -- Movie 2.

**Figure 7:**
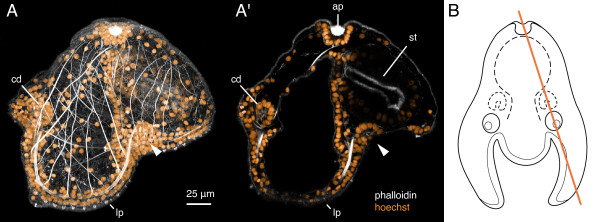
**Trunk disc invaginations in a 10-day-old pilidium of *Micrura alaskensis***. A, A' Confocal images of the pilidium larva stained with phalloidin (white) and hoechst (orange). Apical organ is up, anterior lobe to the left. A. A z-projection of the entire larva, viewed from the left side. The left cephalic disc (cd) and the left trunk disc (arrowhead) are discernible as two dense clusters of nuclei in vicinity of the ciliated band at the anterior and posterior margin of the left lateral lappet (lp). A'. A sub-stack (3.3 μm thick) of the same larva, which highlights the left trunk disc invagination (arrowhead), which originates from subumbrellar pilidial epidermis, i.e. below the ciliated band. The section also passes through the apical organ (ap), the stomach (st), and the left cephalic disc (cd). B. A diagram of the pilidium larva viewed in frontal aspect (apical organ up, anterior lobe and cephalic discs facing us) that illustrates the plane of sectioning (orange line). See also additional file 3 -- Movie 3.

**Figure 8:**
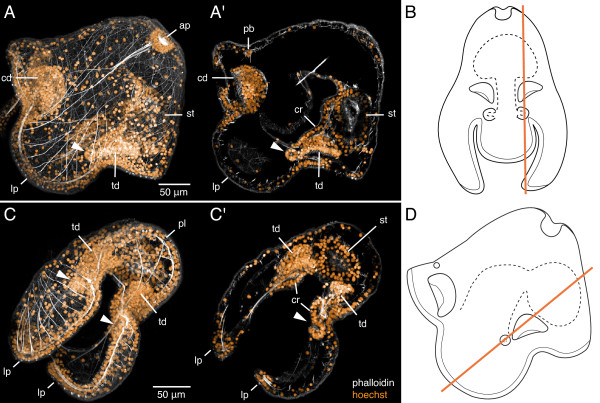
**Cerebral organ disc invaginations in a 17-day-old pilidium of *Micrura alaskensis***. A, A', C, C'. Confocal images of two different pilidium larvae stained with phalloidin (white) and hoechst (orange). A. A z-projection of the entire larva; side view. Apical organ (ap) is at upper right, anterior lobe to the left, stomach (st) to the right. The left cephalic disc (cd), the left cerebral organ disc (arrowhead), and the left trunk disc (td) are discernible as dense clusters of nuclei. A'. A sub-stack (6 μm thick) of the same larva, that highlights the left cerebral organ disc (arrowhead), which originates as an invagination of subumbrellar pilidial epidermis, on the inner side of the lateral lappet (lp) in close proximity to the ciliated ridge (cr). The section also passes through the left trunk disc, the left cephalic disc, the proboscis rudiment (pb), which at this stage already consists of multiple cells, and the stomach. B. A diagram of the pilidium larva viewed in frontal aspect (apical organ up, anterior lobe facing us) that illustrates the plane of sectioning (orange line) for A. Cephalic discs and the proboscis rudiment are omitted for clarity. C. A z-projection of another larva, a slightly oblique view of the space between the lateral lappets and the subumbrellar surface of the posterior lobe (pl). The paired cerebral organ discs (arrowheads) and the trunk discs are discernible as dense clusters of nuclei. C'. A sub-stack (8 μm thick) of the same larva, that highlights the invagination of the right cerebral organ disc (arrowhead). The section also passes through the left and right trunk discs, left and right ciliated ridge, and the stomach. D. A diagram of the pilidium larva in lateral aspect illustrating the plane of sectioning (orange line) for C. See also additional files 4 and 5 -- Movie 4 and Movie 5.

Larvae of *Micrura alaskensis *have three pairs of imaginal discs. The cephalic discs develop first as invaginations of pilidial epidermis just apical of the ciliated band at the junction of pilidial lateral lappets and the anterior lobe - one on each side (Figure 6, Additional file 2 -- Movie 2). The trunk discs develop next as invaginations of the pilidial subumbrellar epidermis (i.e. below the ciliated band), also in the immediate proximity to the larval ciliated band, near the junction of pilidial lateral lappets and the posterior lobe (Figure 7, Additional file 3 -- Movie 3). The last pair, the cerebral organ discs, appear as invaginations of the subumbrellar epidermis on the inner side of lateral lappets in close proximity to the esophageal ciliated ridges and the trunk discs (Figure 8, Additional files 4 and 5 -- Movies 4 and 5).

Once the invaginated rudiments of cephalic and trunk discs separate from the larval epidermis, they consist of two layers separated by a narrow cavity, like a flattened balloon. The thick inner layer gives rise to the juvenile tissues, including the definitive epidermis, and the thin outer layer composed of a single layer of squamous cells gives rise to the amniotic sack, or amnion. The amnion produced by the fused thin outer walls of cephalic and trunk imaginal discs encloses the juvenile inside the pilidium larva and separates it from the blastocoel. In *Micrura alaskensis *the amnion is characteristically decorated with black and brown pigment granules (Figures 3E, G-I, 4A, C, F-H, 5), which become apparent even before the imaginal discs fuse, and are evident in metamorphosing larvae. The black pigment granules are concentrated along the ventral side of the amnion, while the brown pigment granules form a polka-dot pattern on the lateral sides.

**Figure 9:**
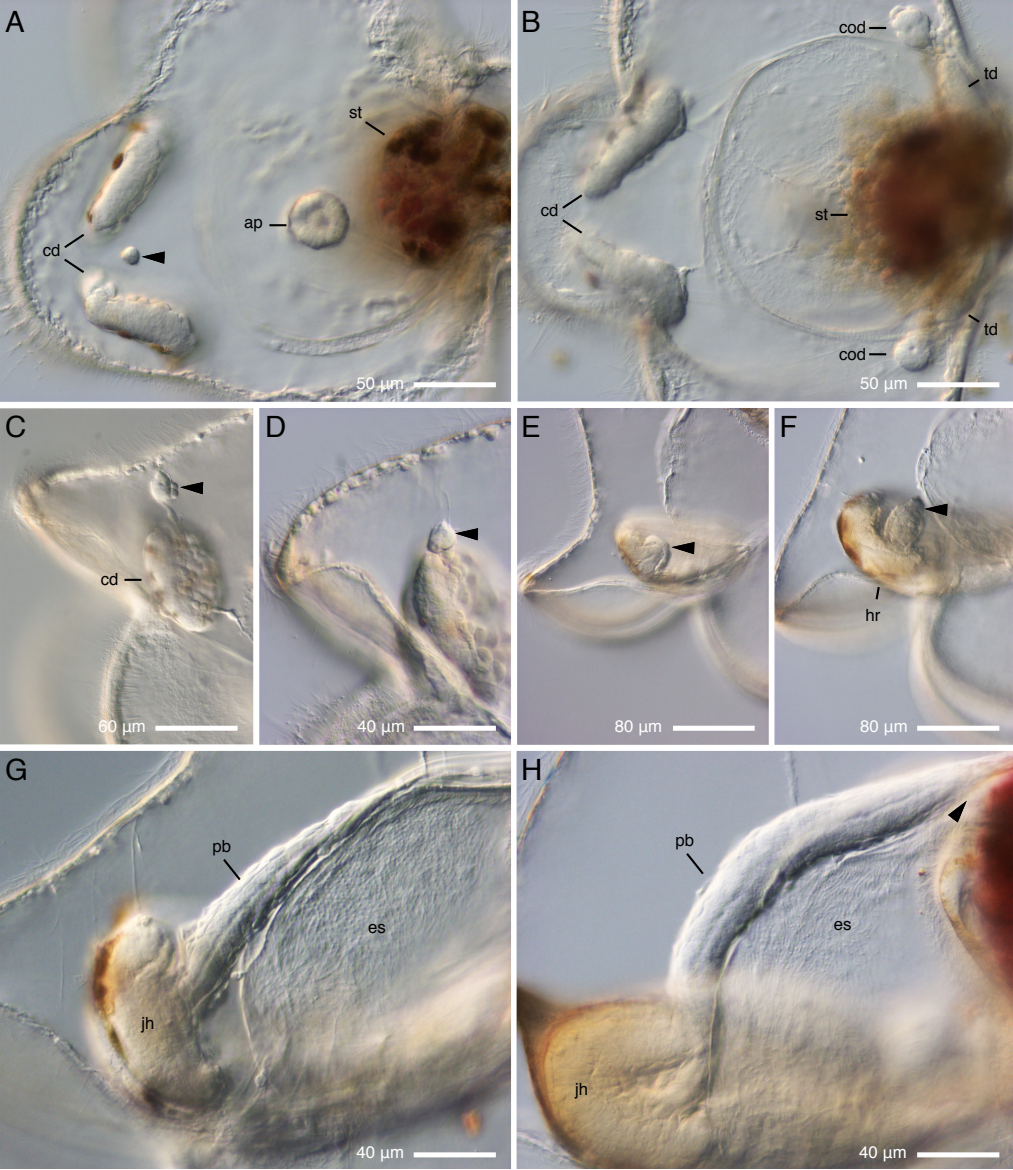
**Development of the proboscis in *Micrura alaskensis***. DIC images. A--B. A pilidium larva viewed from the apical plate (ap), anterior lobe to the left, stomach (st) to the right. C-H. A developmental series showing lateral views of different larvae, apical plate up, anterior lobe to the left. A. Proboscis rudiment (arrowhead) first appears as a small and discrete cluster of cells in the plane of bilateral symmetry of the larva between the two cephalic discs (cd). B. Same larva as on A, a different focal plane, that passes through the paired cerebral organ discs (cod) and trunk discs (td). C. Similar developmental stage as on A: the proboscis rudiment (arrowhead) is separate from the cephalic discs. D. Subsequent developmental stage at which the proboscis rudiment (arrowhead) fuses with the cephalic discs. E. The proboscis rudiment (arrowhead) grows out of the head rudiment as a distinctly bilayered bud. D. The proboscis bud (arrowhead) reaches the upper margin of head rudiment (hr). G. The proboscis (pb) continues to elongate along the larval esophagus (es) and reaches beyond the margin of the juvenile head (jh) at extended proboscis stage. H. The distal tip of proboscis (arrowhead) reaches the stomach (dark magenta shape) at complete proboscis stage.

In addition to the six imaginal discs described above larvae of *Micrura alaskensis *possess two unpaired rudiments -- the proboscis rudiment and the dorsal rudiment. These do not form as epidermal invaginations, but appear to be mesenchymal in origin. The proboscis rudiment is first evident as a distinct small cluster of few cells in the plane of bilateral symmetry of the larva -- between the pilidial epidermis of the anterior lobe and the two cephalic discs (Figures 8A', D, 9A, C). All three pairs of imaginal discs are present at this stage (Figures 8, 9B). Subsequently, the proboscis rudiment fuses with the two cephalic discs (Figure 9D), and forms a distinctly bilayered proboscis bud inside the head rudiment at the "head and trunk" stage (Figure 9E). The proboscis bud elongates (Figure 9F) and protrudes beyond the margin of the head rudiment at "extended proboscis" stage (Figure 9G). The proboscis follows along the larval esophagus in the larval mid-plane and reaches the larval stomach at "complete proboscis" stage (Figure 9H).

The dorsal rudiment, like the proboscis rudiment, is first evident as a cluster of what seems to be few mesenchymal cells in the plane of bilateral symmetry, at the diametrically opposite end from the proboscis. It is sandwiched between the epidermis of the posterior lobe and the stomach (Figure 3F). It subsequently fuses with the trunk discs to form the trunk rudiment (Figure 3G).

### Larval and juvenile serotonergic nervous system

**Figure 10:**
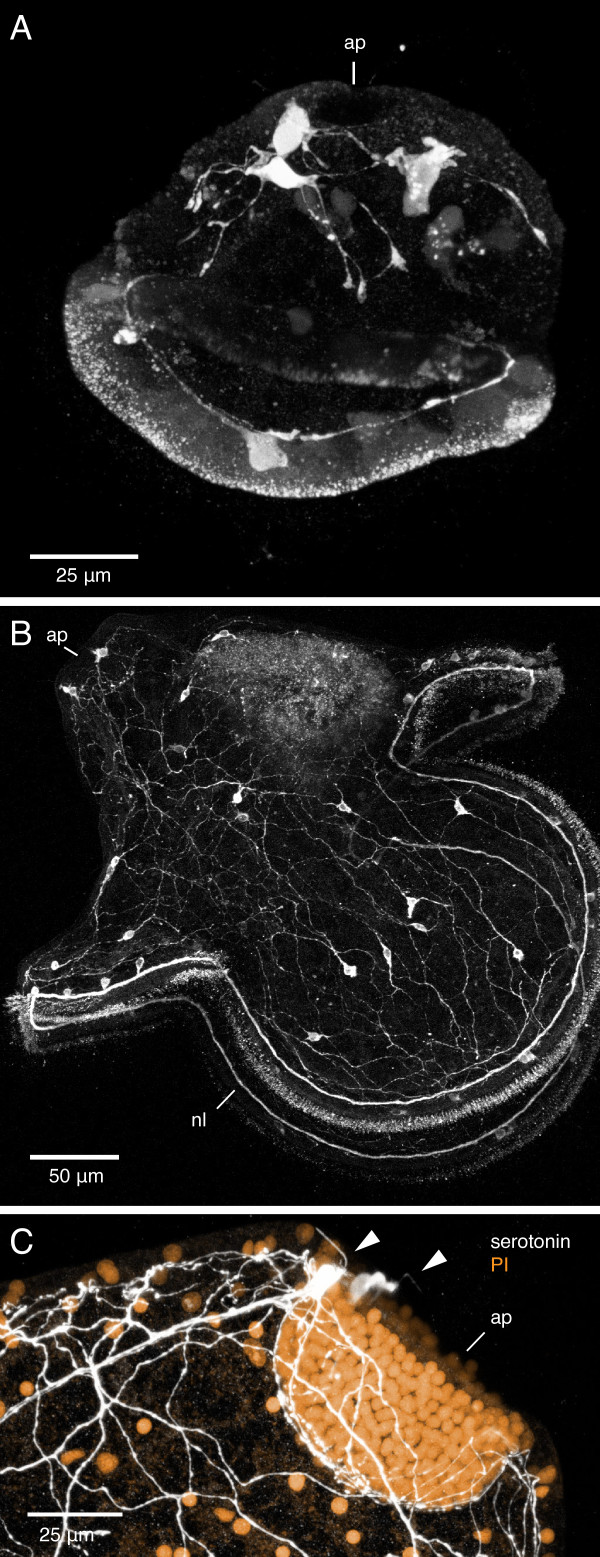
**Serotonergic nervous system of the pilidium larva of *Micrura alaskensis***. Confocal projections. A. Z-projection of a 40-hour-old pilidium larva stained with anti-5HT antibody. Apical plate (ap) is up. Serotonergic neurons are present both in the apical region and along the ciliated band. B. Z-projection of a month-old pilidium larva (head and trunk stage) stained with anti-5HT antibody. Note the extensive serotonergic network, a pair of neurons associated with the apical plate, and the marginal ciliary nerve (nl) associated with the larval ciliated band. C. Z-projection of the apical plate of a month-old pilidium larva stained with anti-5HT antibody (white) and propidium iodide (orange). Note the two serotonergic neurons (arrowheads) associated with the apical plate.

**Figure 11:**
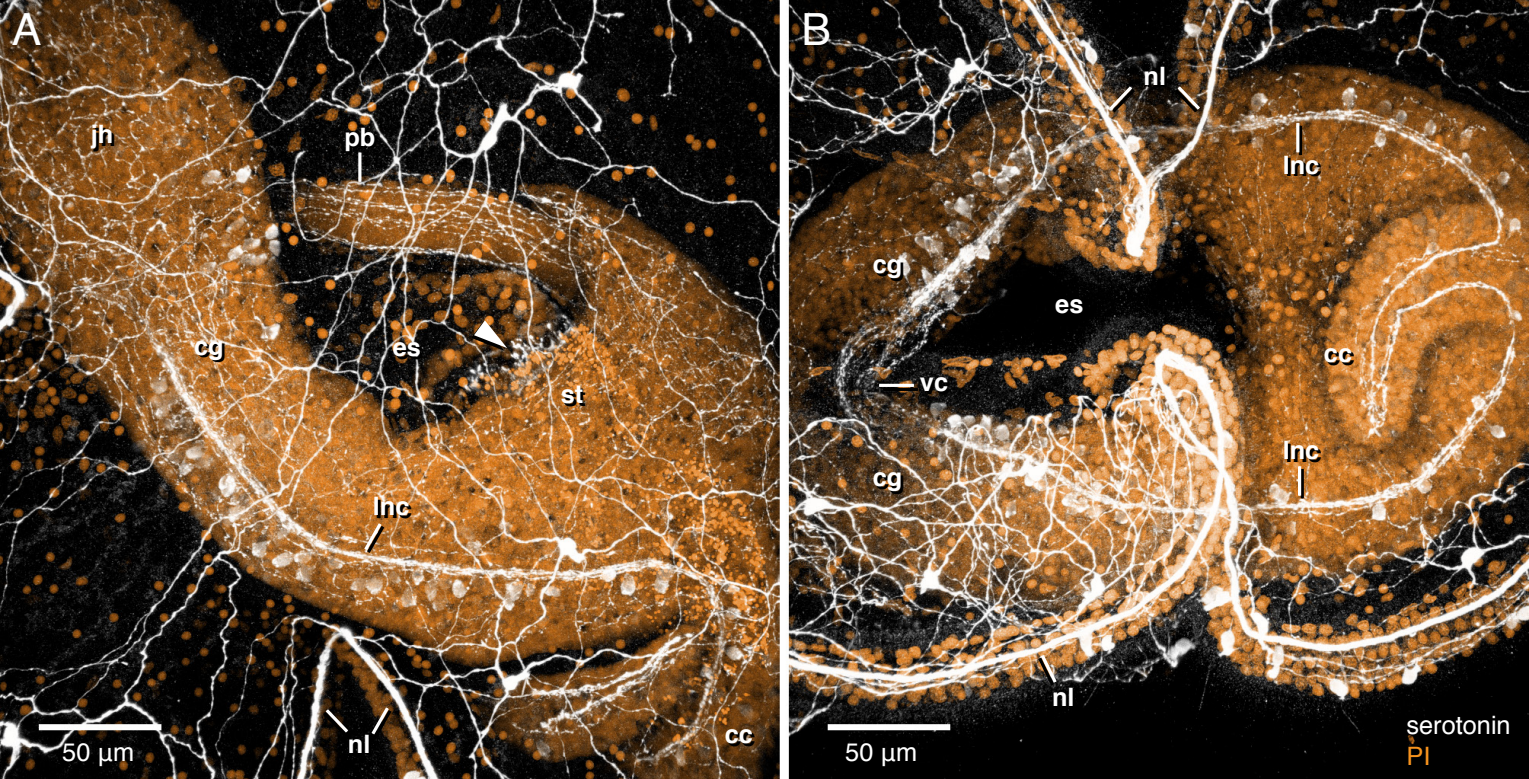
**Serotonergic nervous system of the juvenile inside the pilidium of *Micrura alaskensis***. Confocal z-projections of the juvenile inside the pilidium larva at "complete proboscis" stage of development, stained with anti-5HT antibody (white) and propidium iodide (orange). A. Lateral view, juvenile head (jh) at upper left, juvenile caudal cirrus (cc) at bottom right. A fragment of the marginal ciliary nerve (nl) associated with the larval ciliated band is visible at the bottom center. Juvenile serotonergic nervous system includes paired lateral nerve cords (lnc) which originate from the cerebral ganglia (cg) and join in the caudal cirrus, subepidermal neurite network, longitudinal nerve fibers in the proboscis (pb), and the oral nerve (arrowhead) associated with the sphincter between the larval esophagus (es) and the stomach (st). B. Ventral view, juvenile head (jh) to the left, juvenile caudal cirrus (cc) to the right. Fragments of the marginal ciliary nerve (nl) associated with larval ciliated band are visible at the top and bottom. The juvenile lateral nerve cords (lnc) brightly labeled with anti-5HT antibody connect to the paired cerebral ganglia (cg), which are joined by a thick ventral brain commissure (vc). Lateral nerve cords join at the posterior end as they loop through the caudal cirrus (cc), thus forming a torus around the larval esophagus (es). See also additional files 6 and 7 -- Movie 6 and Movie 7.

Serotonergic neurons were observed in the young pilidium larva as early as 40 hours after fertilization, even before onset of feeding. Two groups of neurons can be distinguished -- several apical neurons, and several associated with the developing ciliated band (Figure 10A). The serotonergic nervous system of a fully developed pilidium larva includes numerous serotonergic neurons connected by the marginal ciliary nerve (terminology after Lacalli and West [31]) associated with the ciliated band, as well as numerous neurons that form an extensive subepidermal nerve net (Figure 10B). Two monociliated serotonergic neurons are always found associated with the apical plate, one on each side (Figure 10B-C). In addition, one can distinguish the circular oral nerve [31] in the sphincter between the larval esophagus and the stomach (Figure 11).

The juvenile inside the pilidium possesses a separate serotonergic nervous system, which becomes evident after torus stage. As in adult nemerteans, the most prominent component of the juvenile nervous system includes the two lateral nerve cords that originate from the cerebral ganglia and are connected anteriorly via dorsal and ventral brain commissures and posteriorly in the juvenile caudal cirrus (Figure 11). In addition, one can distinguish the juvenile subepidermal nerve network, and a number of longitudinal nerves in the proboscis (Figure 11). The only component of the larval serotonergic nervous system that appears to be incorporated into the juvenile is the oral nerve.

**Figure 12:**
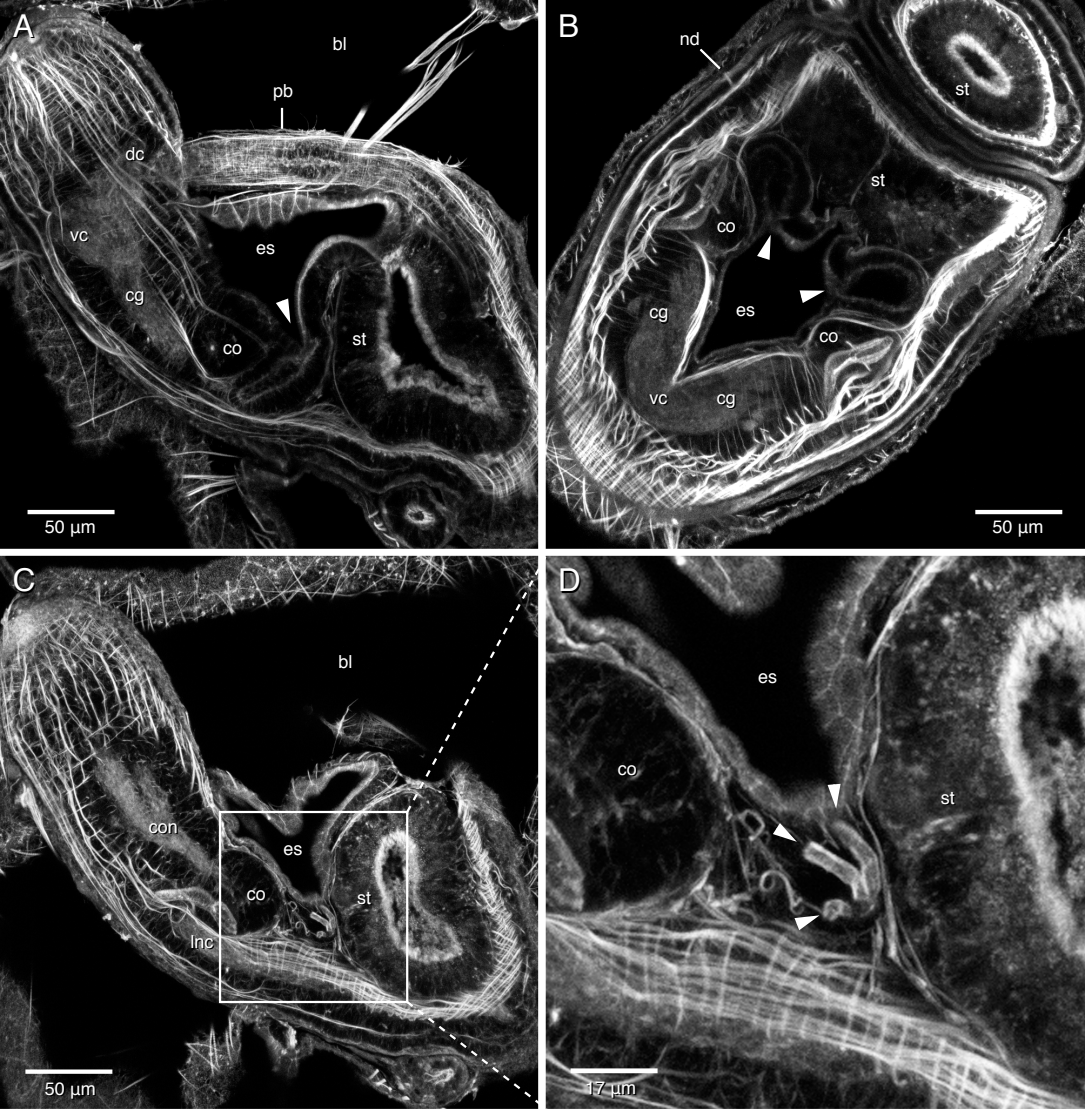
**Esophageal pouches which give rise to the juvenile foregut, and provisional nephridia in *Micrura alaskensis***. Confocal sub-stacks of the juvenile inside the pilidium larva between "complete proboscis" and "hood" stage, stained with phalloidin; juvenile anterior at upper left on A, C and D, and at bottom left on B. A. A z-projection of thirteen 1-μm mid-sagittal confocal sections showing one of the thick-walled pouches (arrowhead) of larval esophagus (es) sandwiched between the cerebral organ (co) and the larval stomach (st). This stack also shows one of the cerebral ganglia (cg), ventral commissure of the brain (vc), and dorsal commissure of the brain (dc). The space dorsal to the proboscis (pb) is the blastocoel (bl), which is traversed by the muscle-retractor of the apical organ (upper right corner). B. A z-projection of ten 1-μm frontal sections through a different larva at a similar developmental stage, showing both of the bilaterally symmetrical esophageal pouches (arrowheads) between the two cerebral organs and the larval stomach. The stack also shows the ventral commissure of the brain connecting the two cerebral ganglia, and one of the nephridioducts (nd). C. Same specimen as on A; a z-projection of ten 1-μm sections slightly more lateral compared to those on A. This stack passes through the cerebral organ (co), cerebral organ canal, the thick nerve (con) which connects the cerebral organ to the cerebral ganglion, and one of the provisional nephridia (in the center of boxed area) sandwiched between the larval esophagus, the larval stomach, and the cerebral organ. D. The boxed area in C magnified to show the three finger-like branches (arrowheads) of the nephridium. See also additional video files 6 and 7 -- Movie 6 and Movie 7.

In addition to serotonergic neurons and fibers, I was able to visualize the fibrous core (axons) or the major components of the juvenile nervous system, such as the cerebral ganglia, the lateral nerve cords, dorsal and ventral commissures of the brain, and the two cerebral organ nerves, using phalloidin labeling (Figure 12A-C, Additional files 6 and 7 -- Movies 6 and 7). From this it is clear that the serotonergic neurons comprise only a small fraction of the juvenile nervous system.

## Discussion

### Development of the paired imaginal discs

The results of this study confirm classical reports that the typical pilidium larva possesses three pairs of imaginal discs which develop as ectodermal invaginations -- the cephalic discs, the trunk discs, and the cerebral organ discs. This study disagrees with the only other detailed report on the order of appearance and the origin of these discs in pilidium larvae [23], and confirms Schmidt's report for *Cerebratulus marginatus *[24,25]. Salensky, who relied solely on planktonic samples reported that all three pairs of imaginal discs appear simultaneously [[23]: p. 21]. Schmidt [24,25] reared pilidia of *C. marginatus *from fertilization to the point of formation of almost all of the juvenile rudiments and reported that the discs appear in the following sequence: cephalic discs first, followed by the trunk discs, and finally, the cerebral organ discs. The present study shows that in *M. alaskensis*, the appearance of the paired imaginal discs follows the same order: the cephalic discs appear after about one week of development, followed by the trunk discs at two weeks, and the cerebral organ discs at about three weeks of development at ambient sea temperature.

My unpublished observations of development in another Northwest Pacific coast species *Cerebratulus *cf. *marginatus *(which is likely not the same species as Schmidt's *C. marginatus *from the Gulf of Naples, Italy), as well as in numerous pilidia belonging to different species captured in plankton in coastal waters of Washington and Oregon suggest that they all follow the same sequence of appearance of imaginal discs. Although the development of the juvenile was not the focus of his study, Cantell's observations of pilidium larvae of *Lineus albocinctus, Lineus bilineatus, Micrura purpurea*, and several unidentified pilidial morphotypes collected from plankton [12] suggest the same order of imaginal disc formation as described by Schmidt [24,25] and in this study. It is easy to see, however, that if one is relying solely on the larvae obtained from plankton, as opposed to rearing them in the lab, one might be misled into thinking that all three pairs of imaginal discs appear simultaneously, if intermediate stages of development did not present themselves to the collector.

**Figure 13:**
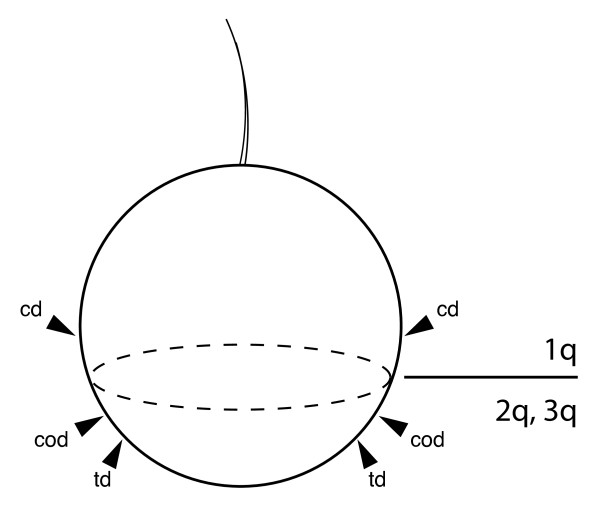
**Origin of the paired pilidial imaginal discs with respect to the larval ciliated band**. This simplified diagram depicts a pilidium larva as a sphere, with the apical tuft pointing up and the subequatorial larval ciliated band represented by a dash line. The cephalic imaginal discs (cd) invaginate from the larval episphere, i.e. above the larval ciliated band, and have been shown experimentally to be derived from the first-quartet micromeres (1q cells). The trunk discs (td) and the cerebral organ discs (cod) invaginate from the hyposphere, i.e. below the larval ciliated band, and therefore are derived either from the second-quartet (2q cells), or the third-quartet micromeres (3q cells).

According to Salensky [23], all three pairs of imaginal discs develop as invaginations of subumbrellar pilidial epidermis, i.e. below the larval ciliated band, or in other words -- from the hyposphere. I document here for *M. alaskensis *that while the trunk discs and the cerebral organ discs indeed invaginate from the subumbrellar epidermis, the cephalic discs are derived from the umbrellar epidermis, i.e. above the larval ciliated band, or from the episphere (Figure 13). This particular observation is in agreement with the known cell lineage of the cephalic discs, which are derived from the first-quartet micromeres in pilidium larvae of *Cerebratulus lacteus *[[32], reviewed in [33]]. The cell lineage of the trunk discs and the cerebral organ discs remains to be determined using long-term lineage markers, but their position suggests that they are produced by the progeny of the second-quartet or the third-quartet micromeres.

### The proboscis rudiment in pilidial development

According to textbooks, the juvenile inside the pilidium larva develops from seven imaginal discs: the paired cephalic discs, paired trunk discs, paired cerebral organ discs, and an unpaired dorsal disc [1,9,30]. This is largely based on the most detailed published study of development of the juvenile inside the pilidium larva by Salensky [23]. Eight imaginal discs, including a separate proboscis rudiment are described in development of Desor's larva [26-29]. According to Salensky [23], the proboscis of the pilidium larva develops from the fused cephalic discs, and a separate proboscis rudiment is lacking. Interestingly, Bürger [22] and Schmidt [24,25] described a separate proboscis rudiment in development of the pilidium larva, but this observation was not confirmed by Salensky [23] and is ignored in the more recent literature.

The results of the present study clearly show that in the pilidium larva of *Micrura alaskensis *there are eight separate juvenile rudiments, including an unpaired proboscis rudiment, which confirms earlier reports by Bürger for an unidentified species [22] and Schmidt for *Cerebratulus marginatus *[24,25]. Because the proboscis rudiment is relatively small and is evident as a separate entity from the cephalic discs only for a short period of time, it is possible that Salensky and other investigators relying as they did on planktonic samples instead of culturing larvae, did not have the opportunity to observe and confirm its presence.

The proboscis rudiment in *M. alaskensis *does not develop as an invagination, contrary to Bürger's report [22]. It is not clear whether it arises by delamination from the pilidial epidermis, or from a cluster of mesodermal cells associated with the pilidial epidermis. In the first case the proboscis rudiment would be derived from the first-quartet micromeres. In the latter it would most likely originate from the 4d cell, the spiralian mesentoblast. The 4d cell gives rise to a population of scattered mesenchyme cells in the pilidium of *Cerebratulus lacteus *[32], including a small cluster of cells in vicinity of the cephalic discs, i.e. where proboscis rudiment would be developing later (Figure 7d in [32]). It appears very likely that the bilayered proboscis bud described here (Figure 6E) has a dual origin, so that its inner part ("the arm") is derived from the fused cephalic discs, as proposed by Salensky [23], while the outer part ("the sleeve") from the separate proboscis rudiment described here. Conceivably, the separate rudiment is derived from the 4d cell and gives rise to the muscle layers of the proboscis and the rhynchocoel, while the portion derived from the cephalic discs gives rise to the glandular epidermis of the proboscis. This would make sense in the context of a now widely accepted hypothesis that the nemertean rhynchocoel is homologous to the annelid and mollusk coeloms [34], which are also derived from the 4d cell. A study utilizing long-lasting cell lineage markers is necessary to determine which is the case.

Another piece of evidence that the proboscis is not derived solely from the cephalic discs, as suggested by Salensky [23], comes from an incidental observation of development of one particular cohort of pilidium larvae reared in the lab, which exhibited numerous abnormalities in juvenile, but not larval, development [Maslakova unpublished]. In many of these pilidia one or several juvenile rudiments were missing, while others appeared to be unaffected. Some larvae had all of the discs, including both of the cephalic discs, but were missing the proboscis. This suggests that even if cephalic discs normally contribute to the proboscis, it is possible that they may need some sort of inductive signal from the unpaired proboscis rudiment in order to do so. It would be possible to test whether the anterior unpaired rudiment observed in this study is essential to the formation of the proboscis by experimentally destroying it (e.g. by laser ablation) in otherwise normally developing pilidium larvae.

### Origin of the dorsal rudiment

Although textbooks often describe all of the juvenile rudiments as invaginations of larval epidermis, the results of this study suggest that only the paired imaginal discs develop as distinct invaginations, whereas the proboscis rudiment and the dorsal rudiment do not. Salensky [23] reported that the unpaired dorsal disc develops via delamination from the pilidial dorsal epidermis. The present study is inconclusive as to whether the dorsal disc is of mesodermal origin (i.e. likely derived from the 4d cell) or is in fact an epidermal derivative (i.e. likely derived from one of the first-quartet micromeres).

### Nephridial rudiments and the juvenile foregut

Several earlier studies describe a pair of esophageal pouches during juvenile development inside Desor's larva [26] and pilidium larvae [22,23] as rudiments of the nephridia (although Nusbaum and Oxner [27] disagree with this interpretation). Bürger [22] reported that the two pouches completely separate from the esophageal cavity of the pilidium larva, fuse with the trunk discs, then branch like fingers of a glove, and that the nephridiopores develop after metamorphosis. Salensky [23] reported two pouches in a similar position (between the cerebral organ discs and the larval stomach), but interpreted them as being derived from the subumbrellar epidermis near esophagus, rather than the esophageal wall. He insisted on this particular distinction, because he believed that the pilidial esophagus is of endodermal origin, and the nephridia must be derived from the ectoderm. Histologically, however, there is no difference between the subumbrellar epidermis and esophageal epidermis; one gradually transitions into the other. Both are composed of squamous cells, and both are likely ingested by the juvenile during pilidial metamorphosis. Moreover, cell lineage analysis in the pilidium larva of *Cerebratulus lacteus *[32] shows that the larval esophagus is derived from the second-quartet and third-quartet micromeres, i.e. is of ectodermal origin. More importantly, however, Salensky [23] did not observe these pouches closing off from the esophagus, even in the latest stages of juvenile formation inside the pilidium larva, nor did he observe any branching.

**Figure 14:**
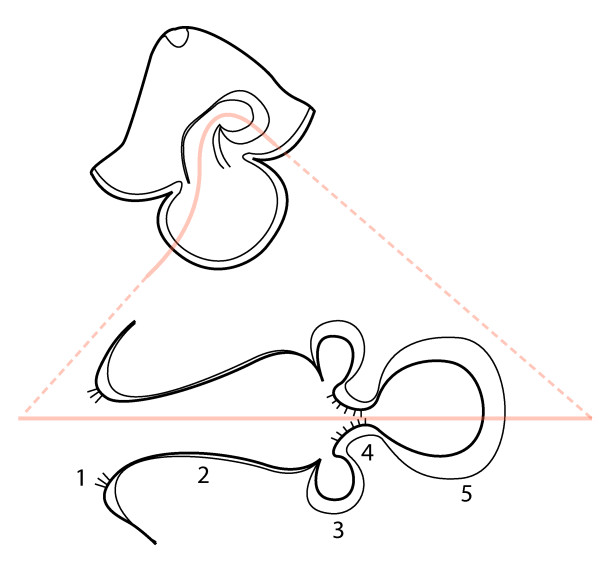
**Relative position of the juvenile foregut rudiments in the pilidial digestive tract**. The lower drawing represents the pilidial digestive tract as if it were straightened along the path of a hypothetical food particle (pink line in each), which passes: 1 - larval ciliated band, 2 - larval esophagus, 3 - esophageal pouches (paired rudiments of the juvenile foregut), 4 - ciliated ridges in the larval esophagus, 5 - stomach.

I observed a pair of thick-walled rounded esophageal pouches sandwiched between the cerebral organs and the larval stomach in advanced developmental stages (e.g. "complete proboscis" and "hood") of *M. alaskensis *(Figure 12, Additional files 6 and 7 -- Movies 6 and 7). Similar to Salensky [23], I did not observe these pouches closing off from the esophagus, or their distal ends branching. Judging from their position and morphology, it seems unlikely that they represent rudiments of nephridia (see also [27]). A more likely explanation is that they form the juvenile foregut, while the larval stomach gives rise to the juvenile midgut. In adult pilidiophorans, these two regions of the digestive tract are well differentiated histologically, and the transition between the two is very distinct. The adult foregut comprises a muscular, thick-walled tube of densely ciliated, glandular, and typically deeply folded epithelium. It appears unlikely that it could be derived from the larval esophagus, or the larval stomach, as previously suggested ([23] and references therein). I observed a distinctly bipartite digestive tract in newly metamorphosed juveniles of *M. alaskensis *with a thick-walled foregut positioned between the cerebral organs and the midgut, where the paired pouches used to be (data not shown). Upon request from one of the reviewers I am including a diagram, which shows relative position of the juvenile foregut rudiments to the various parts of the larval digestive system (Figure 14).

At the same time, I have also observed what appears to be a pair of protonephridia -- one on each side of the developing juvenile, in the immediate vicinity of the paired esophageal pouches described above, but not physically connected to them. These organs, provisionally referred to as the nephridia, are sandwiched between the cerebral organs and the stomach on two sides, and the esophageal pouches and the juvenile body wall on two other sides. Each nephridium has a branched distal portion, and at least one efferent duct leading through the body wall to the nephridiopore on the lateral side of the developing juvenile in advanced stages of *M. alaskensis *(Figure 12, Additional file 7). The position of these rudiments corresponds to that of protonephridia in adult nemerteans (restricted to the foregut region in most species). Because the nephridial rudiments are located in close proximity to the foregut rudiments, and because Bürger's study [22] was based on regular histological sections which are typically 7-8 μm thick (compared to 1-μm-thick confocal sections in this study), he might have been mislead into thinking that a) the nephridial rudiments are connected to the esophageal pouches, b) these pouches are closed off from the esophagus, and c) the nephridial openings develop only after metamorphosis. Future studies utilizing TEM of ultra-thin sections of advanced developmental stages of pilidium larvae should be able to confirm the nature of these provisional nephridia.

### Larval and juvenile nervous systems

The results of this study largely confirm previous reports on the structure of the pilidial larval and juvenile nervous systems based on classical histological methods [22,23], TEM [31] and immunohistochemistry and fluorescent microscopy [35].

Salensky [23] believed that the central nervous system of the juvenile (cerebral ganglia and lateral nerve cords) is derived from the cephalic imaginal discs, that there are no rudiments of the nervous system in the trunk discs, and that the lateral nerve cords invade the tissue of the trunk discs after fusion with the cephalic discs. Bürger [22] believed that the central nervous system is derived from both the cephalic imaginal discs and the trunk discs and that the juvenile nervous system develops at about the time when the cephalic and the trunk discs fuse with each other. According to Bürger [22] the cephalic discs give rise to the dorsal cerebral ganglia, while the trunk discs give rise to the ventral cerebral ganglia and the lateral nerve cords. The results of this study cannot confirm or disconfirm either hypothesis. It does appear very likely that at least some portion of the cerebral ganglia originates from the cephalic discs. However, a long-term lineage tracing is necessary to determine with confidence which imaginal discs give rise to various parts of the juvenile nervous system.

## Conclusions

This study represents the first report documenting pilidial larval development from fertilization to metamorphosis. It is based on observations of laboratory cultures of the common Northwest Pacific coast species *Micrura alaskensis*. Larvae typically reach metamorphosis after 5-8 weeks of development at ambient sea temperature. During metamorphosis the juvenile worm escapes from the larval enclosure and routinely devours the entire pilidial body. The larval and juvenile serotonergic nervous system is for the first time illustrated using confocal microscopy.

Larval development is asynchronous in culture, so a staging scheme is proposed based on certain developmental milestones to facilitate comparison with other species. Characteristic developmental stages include, in that order: blastosquare, gastrula, young pilidium, feeding pilidium, cephalic discs, trunk discs, cerebral organ discs, head and trunk, torus, extended proboscis, complete proboscis, hood, metamorphosis. The order of appearance of juvenile rudiments is always the same and appears to be conserved between different pilidiophoran species.

The paired cephalic discs, paired trunk discs, and paired cerebral organ discs develop as invaginations of the larval epidermis. The cephalic discs invaginate from the larval episphere, while the trunk discs and the cerebral organ discs invaginate from the hyposphere. A separate unpaired dorsal rudiment previously described for pilidium larvae is also found in *M. alaskensis*, and does not develop as an invagination. The rudiments of juvenile nephridia and foregut, which have been described but confused with each other in the earlier literature and ignored by the modern literature are described and illustrated using confocal microscopy. One of the most significant findings of this study is the separate proboscis rudiment, which brings the total number of juvenile rudiments inside the pilidium larva from seven, as described in textbooks, to eight. The unpaired proboscis rudiment does not develop as an invagination, and may be of mesodermal, rather than ectodermal origin.

## Methods

### Collecting adults

**Figure 15:**
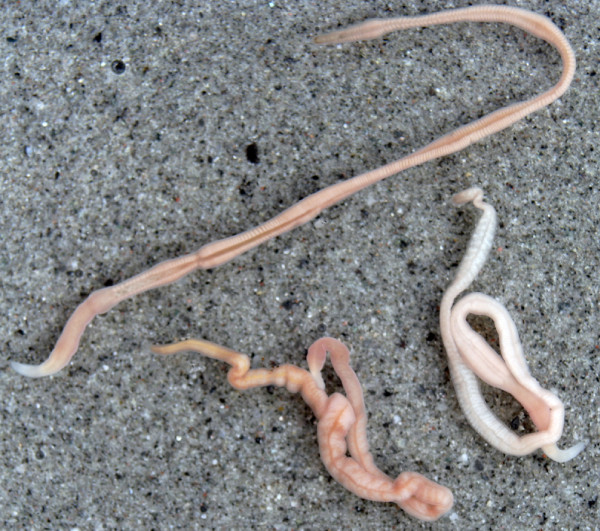
**External appearance of live adults of *Micrura alaskensis***. The worms are about 5-10 cm long and 2-3 mm wide, pinkish with lighter colored head.

*Micrura alaskensis *(Heteronemertea; Lineidae) is a common intertidal pilidiophoran nemertean which inhabits sandy mudflats of the Northwest Pacific coast from Alaska to California (Roe et al 2007). The adults are typically about 5-10 cm long and 2-3 mm wide, light pinkish to darker salmon pink with lighter colored head (Figure 15). *M. alaskensis *lacks ocelli and possesses lateral longitudinal cephalic slits and a caudal cirrus, both easily observed under the dissecting microscope in specimens with intact anterior and posterior end. As in other heteronemerteans, the mouth opens ventrally some distance from the terminal proboscis pore. Adults are usually found in the top 10-20 cm of silty sand and co-occur with nemerteans *Carinoma mutabilis *and *Cerebratulus *spp., polychaetes (Fams. Maldanidae, Onuphidae, Oweniidae, Nereidae, Nephtidae, Lumbrinereidae etc.), phoronids (*Phoronopsis harmeri*) and a variety of sand-burrowing bivalves, such as *Macoma nasuta*, *Clinocardium nuttallii *and *Tresus capax*.

Reproductive adults of *M. alaskensis *were collected during negative low tides in False Bay (San Juan Island, Washington, USA) in July-August 2005, June-August 2006 and July 2007, and from several different mudflats in vicinity of Charleston, Oregon during the summer months of 2008-2010. Occasionally, ripe individuals were encountered outside the usual summer reproductive period, e.g. in February 2007 in False Bay and in March 2009 and March 2010 in Charleston, OR. Ripe males and females can be distinguished under the dissecting microscope, as testis and ovaries are visible through the body wall. Gametes were obtained by dissection. Several hundred to thousands of eggs can be obtained from a single female. Primary oocytes with distinct germinal vesicles dissected from the females or spawned naturally, undergo germinal vesicle breakdown soon upon contact with sea water, and can be fertilized by a dilute suspension of sperm obtained by dissection.

### Larval culture

Developing embryos were cultured in 150 ml glass custard dishes in 0.45 μm-filtered seawater (FSW) until they started swimming. Swimming stages were transferred to larger volumes of FSW and cultured with continuous stirring using plexiglass paddles. Initial concentrations were approximately 1 larva per ml, and they were progressively thinned to about 1 larva per 4-10 ml over the course of several weeks of development. Water was changed by reverse filtration every three to four days, and after each water change larvae were fed either *Rhodomonas lens *at a concentration of 10^4 ^cells/ml, or a mixed diet of 5 × 10^3 ^cells/ml each of *Rhodomonas lens *and *Isochrysis galbana*.

### Light microscopy

Live pilidium larvae were gently trapped between the slide and the coverglass supported by clay feet, and photographed using Nikon Coolpix 4500 digital camera mounted on a Nikon Eclipse 600 equipped with DIC optics or a Leica DCF 400 digital camera mounted on an Olympus BX51 microscope equipped with DIC. The timelapse video of metamorphosis was captured with side illumination through the Nikon Eclipse 600 microscope equipped with a Sony 3CCD video camera.

### Fluorescent labeling and confocal microscopy

Larvae were relaxed in a 1:1 mixture of 0.33 M MgCl_2 _and filtered seawater for 10-15 minutes at room temperature (RT) and fixed for 30 min at RT in 4% paraformaldehyde freshly prepared from 16% ultrapure paraformaldehyde (Electron Microscopy Sciences) and filtered seawater. Following fixation, larvae were either rinsed in three 10 min changes of phosphate buffer saline (PBS) and stored in PBS at 4°C, or immediately permeabilized and stained. For confocal microscopy larvae were permeabilized in three 10 min changes of phosphate buffered saline (PBS) with 0.1% Triton X-100 (PBT), stained with Bodipy FL Phallacidin (1 U per 100 μl of PBT) and a nuclear dye Hoechst 33342 (1 μM) for 40 min at RT, rinsed in three 10 min changes of PBS and mounted in Vectashield (Vector Laboratories) on Poly-L-lysine coated coverslips supported by foil tape over microscope slides. These semi-permanent preps were sealed with nail polish and viewed immediately or stored at 4 degrees C.

To visualize the serotonergic nervous system, larvae were fixed and permeabilized as described above, then blocked in 5% Normal Donkey Serum (Jackson Immunoresearch) in PBT for 2 hours at RT. After three 10 min washes in PBT, larvae were incubated for 2 hours at RT or overnight at 4 degrees C; with the rabbit-anti-5HT primary antibody (ImmunoStar Cat.# 20080) diluted 1:500 in PBT, washed in three 10 min changes of PBT and incubated for 2 hours at RT with Alexa Fluor 488 donkey-anti-rabbit secondary antibody (Molecular Probes) diluted 1:600 in PBT, adding 10 μg/ml of propidium iodide for the last 40 minutes of incubation to stain the nuclei. Stained larvae were washed in three 10 min changes of PBS and mounted as described above.

Stained samples were examined with a BioRad Radiance 2000 laser scanning confocal mounted on a Nikon Eclipse E800 microscope with a 40× 1.3 N.A. oil lens or an Olympus Fluoview 1000 laser scanning confocal mounted on an Olympus IX81 inverted microscope with a UPlanFLN 40× 1.3 NA oil lens or a PlanApoN 60× N.A. 1.42 oil lens. Stacks of 0.5-1 μm optical sections were imported into ImageJ v. 1.42i (Wayne Rasband, National Institutes of Health, Bethesda, MD, USA) for image processing.

## Competing interests

The author declares that they have no competing interests.

## Supplementary Material

Additional file 1 **Movie 1: Metamorphosis of the pilidium larva of *Micrura alaskensis***. This time-lapse movie shows a typical sequence of pilidial metamorphosis, in which the juvenile successively breaks through the amniotic membrane and the larval epidermis, then backs out of the larval body while simultaneously consuming the larval tissues. In this sequence, the entire larval body is devoured within a few minutes, and the young nemertean worm crawls away having finished its first meal. The video is sped up 2.5× compared to real time. Click here for file

Additional file 2 **Movie 2: Cephalic disc invaginations in a 7-day old pilidium larva of *Micrura alaskensis***. A running z-projection movie of the confocal z-series that was used to make Figure 6. Phalloidin is white, nuclear dye hoechst -- orange. Click here for file

Additional file 3 **Movie 3: Trunk disc invaginations in a 10-day old pilidium larva of *Micrura alaskensis***. A running z-projection movie of the confocal z-series that was used to make Figure 7. Phalloidin is white, nuclear dye hoechst -- orange. Click here for file

Additional file 4 **Movie 4: Three pairs of imaginal discs and the proboscis rudiment in a 17-day old pilidium larva of *Micrura alaskensis***. A running z-projection movie of the confocal z-series that was used to make Figures 8A and 8A'. Phalloidin is white, nuclear dye hoechst -- orange. Click here for file

Additional file 5 **Movie 5: Cerebral organ disc invaginations in a 17-day old pilidium larva of *Micrura alaskensis***. A running z-projection movie of the confocal z-series that was used to make Figures 8C and 8C'. Phalloidin is white, nuclear dye hoechst -- orange. Click here for file

Additional file 6 **Movie 6: Juvenile foregut invaginations in a "complete proboscis" stage of *Micrura alaskensis***. A running z-projection movie of the confocal z-series that was used to make Figure 12B. Frontal sections. Phalloidin labeling. Click here for file

Additional file 7 **Movie 7: Juvenile foregut invaginations and nephridia in a "complete proboscis" stage of *Micrura alaskensis***. A running z-projection movie of the confocal z-series that was used to make Figures 12A,12C, and 12D. Sagittal sections. Phalloidin labeling. Click here for file
